# Glomus Tumor: “A-Not-So-Rare” Cause of Chronic Shoulder Pain—A Case Report and Literature Review

**DOI:** 10.1155/2021/6666092

**Published:** 2021-05-27

**Authors:** Luís Vieira, Pedro Pereira, Bernardo Nunes, Rui Matos, António Sousa, Manuel Ribeiro da Silva

**Affiliations:** Centro Hospitalar Universitário São João, Portugal

## Abstract

**Case:**

A 61-year-old male presented with chronic shoulder pain resistant to conservative treatment. Imaging identified a nodular lesion in the deltoid muscle, which histology after resection identified as a glomus tumor. After surgery, the patient became asymptomatic and at 4-year follow-up has not shown signs of recurrence.

**Conclusion:**

Glomus tumors around the shoulder should be considered when investigating chronic shoulder pain, as they are more common than thought. Despite being elusive, when diagnosed, excellent outcomes may be expected, with surgery resection being curative.

## 1. Introduction

Glomus tumors are benign soft tissue neoplasms of vascular origin [1]. These tumors are classically located under the fingernail beds [2], but extradigital locations are being reported more frequently [3]. While still uncommon, atypical locations of glomus tumors may be debilitating injuries and prove to be cumbersome for the orthopedic surgeon [4].

In this paperwork, we present a rare case of chronic shoulder pain caused by a glomus tumor in the deltoid muscle. As these lesions appear to be more prevalent than assumed, we also present a compilation of all the published cases and a short literature review.

## 2. Statement of Informed Consent

The patient was informed that data concerning the case would be submitted for publication, to which he consented.

## 3. Case Report

A 61-year-old right-handed male was referred to our department presenting shoulder pain for 12 months, without any known trigger event. The patient described a dull intermittent right shoulder pain, reporting acute exacerbations and tenderness. His complaints persisted despite medical treatment, including physiotherapy and long-standing medication with oral NSAIDs.

During physical examination, there were no visible asymmetries between the shoulders and the patient exhibited no inflammatory signs. The neurologic and vascular examination of the right upper limb was normal. The shoulder range of motion and strength was preserved and painless. There were no evident or palpable masses, but the patient presented trigger-point tenderness at the posterolateral surface of the right shoulder.

**Figure 1:**
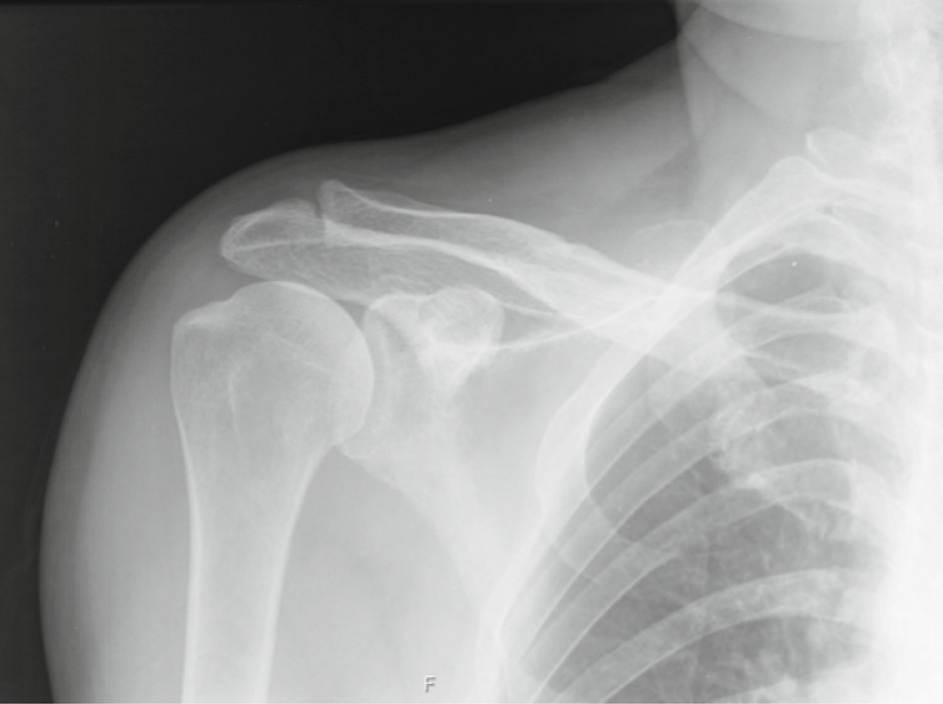
Anteroposterior radiograph of the right shoulder. The radiograph is normal.

**Figure 2:**
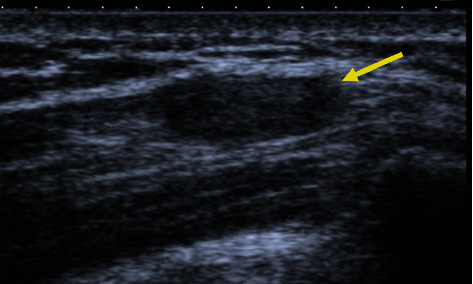
Ultrasonography of the right shoulder identified a hypoechoic nodular formation (yellow arrow) in the posterior deltoid, with approximately 8 × 10 × 13 mm in size.

**Figure 3:**
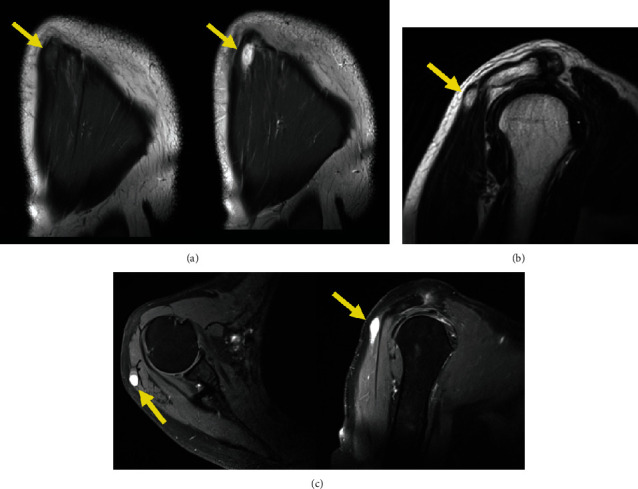
MRI images of the right shoulder. (a) Coronal images showing a nodular formation in the deltoid muscle (yellow arrows). The lesion is hypointense in T1 (left picture), but with strong enhancement with contrast (right picture). (b) Sagittal T2 image with turbo spin echo before contrast. The lesion appears as a hyperintense nodule (yellow arrow). (c) Axial (left) and sagittal (right) T1 images with fat suppression after contrast show a hyperintense well-limited lesion in the posterior deltoid (yellow arrows).

Plain radiographs were normal (Figure 1). Ultrasonography of the right shoulder denoted a hypoechoic nodular formation in the point of tenderness, measuring 13 mm (Figure 2). Magnetic Resonance Imaging (MRI) confirmed the presence of a well-limited oval-shaped mass located in the posterior fibers of the right deltoid muscle, measuring 25 × 10 × 6 mm, which was hypointense in T1 and hyperintense in T2-weighted images and exhibited strong enhancement with contrast (Figures 3).

**Figure 4:**
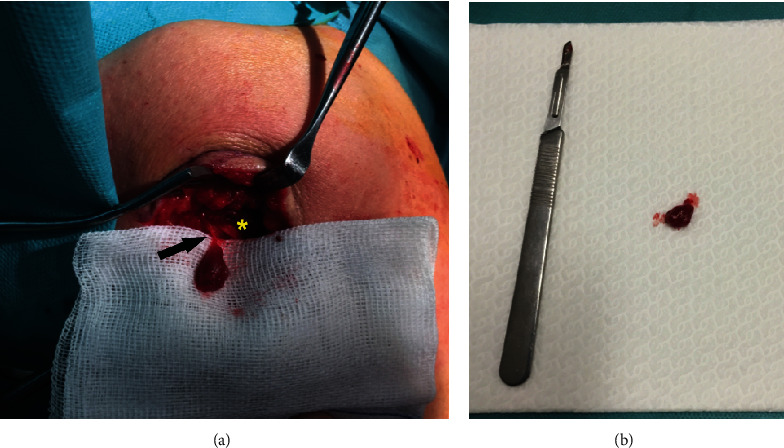
Intraoperative images. (a) Isolated tumor, primarily found in the substance of the deltoid muscle (yellow asterisk), with the tumor pedicle still intact (black arrow). (b) Resected lesion (125 mm scalpel handle with n°11 blade for scale).

It was decided to perform an excisional biopsy of the lesion. Under general anesthesia, the patient was placed in 30 degrees left-lateral position and a 5 cm incision was made directly over the point of tenderness which had been priory marked. Surgical exploration identified a well-limited nodular lesion between the posterior fibers of the deltoid muscle, which was completely excised (Figures 4(a) and 4(b)).

The patient was discharged in the next day after surgery and presented pain-free by the time of stitch removal.

**Figure 5:**
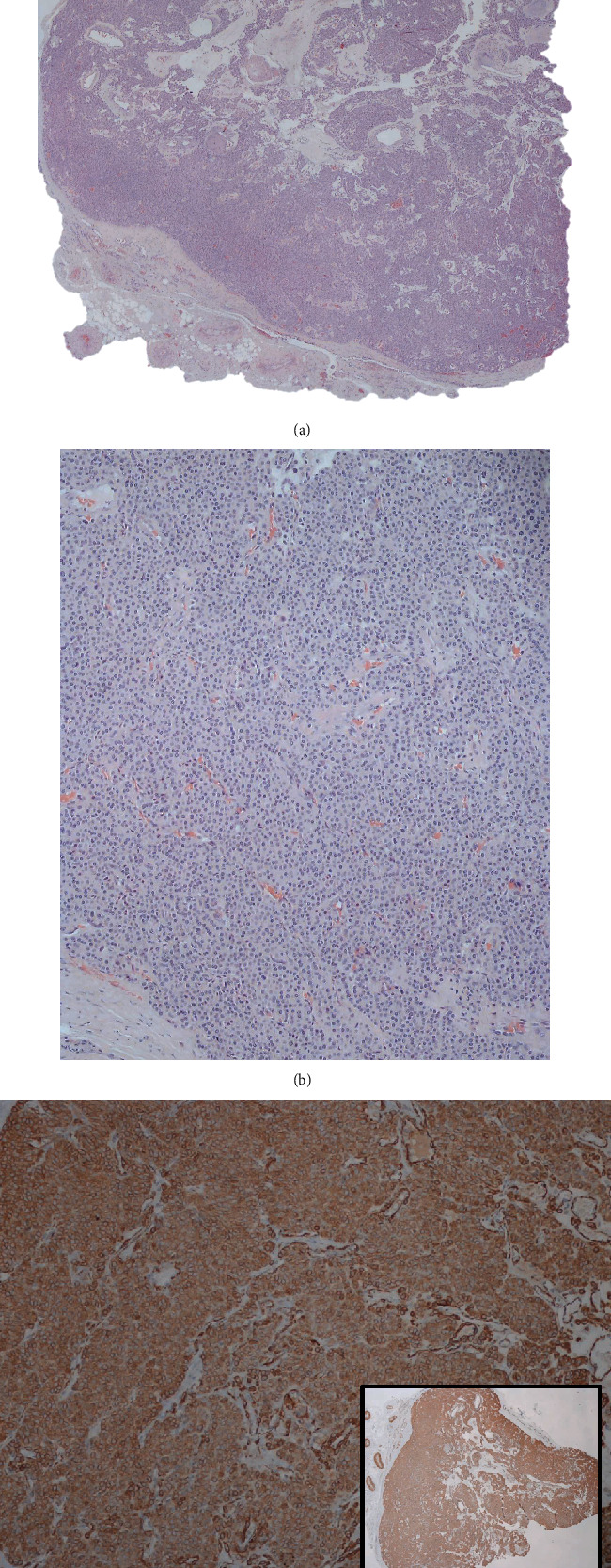
Histological images of the removed tumor. (a) Well-limited proliferative lesion, with a rich vascular network (hematoxylin-eosin; original magnification ×20). (b) Tumor formed by ovoid, epithelial cells, without signs of atypia and showing no mitoses (hematoxylin-eosin; original magnification ×40). (c) Diffuse expression of smooth muscle actin (alpha-SMA) in the tumor (immunohistochemcial staining; original magnification ×20 (right inferior corner photomicrograph) and original magnification ×100 (larger photomicrograph)).

Gross examination of the specimen revealed a brownish nodular mass with 10 × 10 × 6 mm in size, while microscopic examination showed a well-limited lesion formed by ovoid epithelial cells without signs of atypia. No mitoses were observed. The lesion displayed a rich vascular network. Marginal excision was confirmed. Immunohistochemical staining was positive for expression of actin (alpha-SMA). These findings were compatible with the diagnosis of glomus tumor (Figures 5(a)–5(c)).

The patient did not undergo any other treatment besides surgical excision of the lesion.

After 4 years of follow-up, the patient is asymptomatic and showing no clinical or imagiological evidence of local or systemic recurrence.

## 4. Discussion

Glomus tumors are vascular benign neoplasms [1, 5], arising at the glomus bodies, a specialized arteriovenous anastomosis which controls blood flow to the skin and thereby regulating temperature at the extremities [6].

Despite being widely scattered, glomus bodies are far more frequent in the digits, palms, and soles of feet [7], with 70% taking place in the hand and 50% of these arising in the subungual region [2, 8]. Nevertheless, they can be found anywhere in the body [9].

In general, glomus tumors represent 1-6% of all soft tissue tumors [4] and 1-5% of all hand tumors [10]. They seem more prevalent between the third and fifth decades of life and do not seem to show gender predilection [11]; however, subungual locations seem somehow more prevalent in females [1]. Overall, glomus tumors are lonely lesions [4] but may present as multiple tumors in up to 10% of patients [9].

Extradigital locations seem less rare than what was previously believed [3, 4]. Other locations in the upper limb are the most common [4], but there have been reported cases of ectopic glomus tumor in the trunk, lower body, and even viscera [1, 3, 12].

Glomus tumors are generally small lesions (<1 cm in diameter) [12] but particularly around the shoulder can show greater dimensions, with reported cases of lesions as big as 5 cm in diameter [13]. When superficial, they can be identified on inspection as they show a distinctive blue-red coloration [12, 14].

To our knowledge, this is the 4^th^ case of a glomus tumor located in the deltoid muscle [6, 15, 16]. This paper is also currently the most comprehensive compilation of glomus tumors around the shoulder, gathering all known 23 currently published cases in the English literature (Table 1).

Extradigital locations impose true diagnostic challenges [17] and may be responsible for chronic pain and time-consuming investigation [4], often causing distress to the patients who are not uncommonly misdiagnosis with psychiatric illness [3, 14, 18]. In a series by Schiefer et al., glomus tumor was considered in the initial differential diagnosis in only 9% of cases [3]. Concerning the specific location of glomus tumors around the shoulder, the literature has been rich in cases in which diagnosis was delayed for several years, even decades [6, 17, 19–21]. These patients are usually thought to suffer from more common pathologies such as rotator cuff lesions [6], which highlights the need to have a high index of suspicion.

Glomus tumors are classic manifested by the triad of paroxysmal pain, cold hypersensitivity, and pinpoint tenderness, which when present can be considered diagnostic [7]. 63-100% of patients with glomus tumor of the hand exhibited this triad [3], which is fully present in only two cases of tumors around the shoulder [15, 17]. Some patients may present with hyperesthesia, muscle atrophy, or osteopenia of the affected area [4]. In this paper, the patient presented only paroxysms of pain and local tenderness, as did the majority of similar cases published in the literature (Table 1).

Moreover, there have been described several clinical tests which may help in the diagnosis of glomus tumors, such as the pin test of Love, the Posner test (pain induced by cold), the Hildreth ischemia test, and transillumination [6, 22]. Nonetheless, the validity of these tests seems limited to the digits [6].

MRI can be helpful in the diagnosis of glomus tumors, which mostly exhibit low signal intensity on T1-weighted images, hyperintensity on T2-weighted images, and enhancement on T1-weighted images after gadolinium injection [23]. In addition, glomus tumors may display the very typical finding of a high-signal nidus on T2 circumscribed by a low-signal ring, which is not evident in all the cases though [9, 23].

MRI has been described to have a sensitivity of 90% for the diagnosis of glomus tumours [24], being able to detect lesions as small as 2 mm in diameter [23]. Nevertheless, in a setting of high index of clinical suspicion, a negative MRI should not reject the diagnosis [3]. Moreover, the specificity has been reported to be as low as 50% [25], which, associated with the high cost of this exam compared to sonography, settles MRI as a second-line imaging modality [23].

The final diagnosis is confirmed by microscopic and immunohistochemistry examination of the lesion [17]. Histologically, these tumors have been classified accordingly to the predominant expression of glomus cells, blood vessels, and smooth muscle cells in solid glomus tumors (73%), glomangiomas (25%), and glomangiomyomas (8%), respectively [4, 26]. Immunohistochemical analysis reveals vascular and muscular phenotype, with a distinguishing and diffuse expression of *α*-SMA (alpha smooth muscle actin) and a variable expression of CD34 [9].

Surgery is usually required, as conservative treatment is often unsatisfactory [3, 16, 23]. Marginal excision is normally sufficient in the treatment of these neoplasms [12, 27] without the need of adjuvant therapy [13], tough adjuvant radiotherapy may be used in the cases of incomplete resection [17, 27].

Malignization is rare [26]. Of those cases reported around the shoulder, only Rishi et al. have reported a malignant lesion [11]. Folpe et al. have described several features to determine malignancy of these neoplasms: (1) large size (>2 cm), (2) deep location, (3) atypical mitotic figures, (4) moderate to high nuclear grade, and (5) ≥5 mitotic figures/50 HPF [26]. Based on this classification, one could argue that our case should be considered a malignant lesion, as the intramuscular location of the lesion qualifies as a deep location. However, we did not intend to perform a diagnostic biopsy before surgery and we were already aiming for curative treatment with marginal excision. Free margins were confirmed by histology which, furthermore, excluded other aspects of malignancy in the lesion.

Surgery outcome is excellent [3], with prompt disappearance of the symptoms after excision [12]. There are no documented cases of recurrence around the shoulder.

The atypical location, the usual small-size of the lesions, and deeper locations explain why these lesions are frequently overlooked on common imaging exams such as simple ultrasonography, accounting for the delay in diagnosis. In our case, despite being small in size, the lesion was fortunately identified on ultrasonography which prompted further investigation. The fact that full recovery is expected after adequate treatment further emphasis the needs to raise awareness for this pathology when considering causes of chronic shoulder pain.

## 5. Conclusion

Glomus tumors around the shoulder seem less rare than what was previously believed and should therefore be included in the differential diagnosis of unexplained shoulder pain. MRI may help confirm diagnosis in a high suspicious clinical setting. Surgery with marginal excision is usually curative, and excellent clinical outcome is expected.

## Figures and Tables

**Table 1:** Table compiling all cases of glomus tumors around the shoulder published in English literature.

Paper (year published)	Age	Gender	Location	Size (cm)	Time until diagnosis (years)	Symptoms
Bailey (1935) [19]	48	Male	Lateral shoulder	0.3	20.0	Pain, tenderness
Riveros & Pack (1951) [28]	40	Female	Scapular region	0.5	NR	Pain
Massey (1992) [29]	41	Female	Supraescapular region	1.0	Several	Pain, tenderness
Heys et al. (1992) [7]	NR	NR	NR	NR	NR	NR
Calonje and Fletcher (1995) [30]	NA	NA	Cutaneous intraneural	NA	NA	NR
Yoshikawa et al. (1996) [20]	35	Female	Rotator cuff	4.0	20.0	Pain
Roberts et al. (1999) [21]	67	Male	NA	3.5	20.0	Pain, tenderness
Ghaly and Ring (1999) [17]	62	Male	Subcutaneous (supraclavicular)	1.0	20.0	Pain, tenderness, cold
Abela et al. (2000) [14]	52	Male	Subcutaneous	1.5	10.0	Pain, tenderness
Solivetti et al. (2002) [18]	58	Male	NR	0.4	1.0	NR
Schiefer (2016)—2 cases [3]	38/NR	Male/NR	Subcutaneous (medial scapula)/NR	0.6/NR	2.0/NR	Pain, tenderness/NR
Boretto et al. (2008) [6]	54	Female	Insertion deltoid	NR	30.0	Pain, tenderness
Gautam et al. (2008) [31]	25	Female	Acromion	NR	5.0	Pain, tenderness
Karakurum et al. (2009) [15]	71	Male	Posterior fibers deltoid	2.5	0.5	Pain, tenderness, cold
Rishi et al. (2012) [11]	51	Male	Subcutaneous posterior scapula (glomangiosarcoma)	1.0	0.165	Pruritus
Proietti et al. (2013) [4]	30	Female	Subcutaneous posterior scapula	4.0	1.0	Pain, paresthesia
Geramizadeh et al. (2015) [13]	25	Female	Adjacent clavicle	4.9	Since adolescence	Pain, tenderness
Singh et al. (2016) [27]	70	Male	Suprascapular notch	NR	6.0	Pain, tenderness
Beytemür et al. (2016) [16]	68	Male	Anterior fibers deltoid	3.0	1.0	Pain, tenderness
Ravikanth et al. (2018) [32]	56	Male	Subcutaneous (supraclavicular)	<1.0	4.0	Pain, tenderness
Present case	61	Male	Posterior fibers, deltoid	1.0	1.0	Pain, tenderness

NR: nonregistered; NA: not available.

## Data Availability

Data can be available on request. To request the data, please contact the corresponding author: Luís Pedro Vieira (address: Alameda Professor Hernâni Monteiro, Serviço Ortopedia e Traumatologia Centro Hospitalar S. João, 4200 Porto, Portugal; email: luisppvieira@gmail.com).
